# Impact of the Population Medicine Multimorbidity Intervention in Xishui County (POPMIX) on People at High Risk for Chronic Obstructive Pulmonary Disease: Protocol for the POPMIX-COPD Cluster Randomized Controlled Trial

**DOI:** 10.2196/85597

**Published:** 2026-02-18

**Authors:** Chen Wang, Yuhao Liu, Ke Huang, Zhoutao Zheng, Shiyu Zhang, Wenjin Chen, Xingyao Tang, Zhong Cao, Xunliang Tong, Lei Tang, Jinghan Zhao, Liu He, Lirui Jiao, Yingping Wang, Tianying Zhao, Yingchi Luo, Qiande Lai, Xiangqin Lyu, Qiushi Chen, Aditi Bunker, Sebastian Vollmer, Pascal Geldsetzer, Dean Jamison, Till Bärnighausen, Ting Yang, Simiao Chen, POPMIX Group

**Affiliations:** 1 School of Population Medicine and Public Health Chinese Academy of Medical Sciences & Peking Union Medical College Beijing China; 2 Department of Pulmonary and Critical Care Medicine China-Japan Friendship Hospital Beijing China; 3 National Center for Respiratory Medicine Beijing China; 4 State Key Laboratory of Respiratory Health and Multimorbidity Beijing China; 5 School of Health Policy and Management Chinese Academy of Medical Sciences & Peking Union Medical College Beijing China; 6 Heidelberg Institute of Global Health Faculty of Medicine and University Hospital Heidelberg University Heidelberg Germany; 7 Department of Pulmonary and Crtical Care Medicine Beijing Hospital Beijing China; 8 Institute of Geriatric Medicine Chinese Academy of Medical Sciences Beijing China; 9 Guizhou Medical University Guiyang China; 10 Department of Health Policy and Management Gillings School of Global Public Health University of North Carolina at Chapel Hill Chapel Hill, NC United States; 11 Center for Disease Control and Prevention of Xishui County Zunyi China; 12 Department of Pulmonary and Critical Care Medicine People's Hospital of Xishui County Zunyi China; 13 The Harold and Inge Marcus Department of Industrial and Manufacturing Engineering Pennsylvania State University University Park, PA United States; 14 Department of Economics and Centre for Modern Indian Studies University of Göttingen Göttingen Germany; 15 Division of Primary Care and Population Health Department of Medicine Stanford University Stanford, CA United States; 16 Chan Zuckerberg Biohub San Francisco San Francisco, CA United States; 17 Department of Epidemiology and Biostatistics and Institute for Global Health Sciences University of California, San Francisco San Francisco, CA United States; 18 Department of Global and Population Health Harvard T.H. Chan School of Public Health Harvard University Boston, MA United States; 19 See Acknowledgments

**Keywords:** population medicine, multimorbidity, tobacco-related NCDs, high-COPD-risk population, cRCT

## Abstract

**Background:**

Tobacco-related noncommunicable diseases (NCDs) present a major public health challenge in China, requiring population-level management. Chronic obstructive pulmonary disease (COPD) is the most common and prevalent chronic respiratory disease associated with tobacco use. In addition, COPD shares risk factors with other NCDs that frequently co-occur, leading to multimorbidity. This study focuses on the early detection and integrated management of COPD and related multimorbidity among high-risk populations. Population medicine, an emerging and evolving concept aimed at maximizing population health and well-being, provides a promising framework for shifting interventions against COPD from an individual patient focus to a population-level approach.

**Objective:**

This study aims to evaluate the effectiveness of a population medicine–based multimorbidity intervention package among individuals at high risk for COPD.

**Methods:**

We are conducting a 2-arm, population-based, stratified cluster randomized controlled trial (cRCT). The intervention integrates community screening, chronic disease management, patient education, digital follow-up, and team-based care. The trial is being implemented in Xishui County, Guizhou Province, a low-resource county in Southwestern China. Each of the 26 townships in Xishui County was considered a cluster and stratified into large and small townships based on population size. An equal number of residents from each township stratum (large and small) were randomized to undergo the COPD Screening Questionnaire. Individuals identified as being at high risk for COPD were considered study participants and were subsequently enrolled in either the intervention or control arm. The target sample size was approximately 2850 individuals.

**Results:**

Data collection for the POPMIX-COPD trial began in June 2024. Baseline, 3-month, and 6-month assessments have been completed, and 12-month follow-up assessments are planned to be completed in March 2026. All participants in the intervention arm are being followed for 1 year, with 1 telephone follow-up at month 3 and in-person follow-ups at months 6 and 12. Primary outcomes for each participant include the number of chronic conditions controlled, receipt of lung function testing, and forced expiratory volume in 1 second. In addition, secondary outcomes were health-related quality of life, mental and behavioral health status, health care utilization, knowledge of COPD and asthma, and care cascade indicators for chronic conditions.

**Conclusions:**

This cRCT is the first multimorbidity intervention study designed within the population medicine framework to target populations at high risk for COPD. It was featured as a case study in the report of the Lancet Commission on Investing in Health. The results of the trial are expected to inform the next generation of multimorbidity management and population medicine practices among global health authorities and practitioners.

**Trial Registration:**

ClinicalTrials.gov NCT06456996; https://clinicaltrials.gov/ct2/show/NCT06456996

**International Registered Report Identifier (IRRID):**

DERR1-10.2196/85597

## Introduction

The concept of population medicine focuses on maximizing health and well-being at the population level—in contrast to clinical medicine’s traditional focus on the individual patient—and has been gaining attention in recent literature [1]. Population medicine is a progressive medical discipline that aims to promote health equity through the following mechanisms: (1) integrating clinical medicine and public health; (2) providing comprehensive care that encompasses health promotion, disease prevention, diagnosis, control, treatment, and rehabilitation; (3) integrating and applying the knowledge, academic principles, and technologies of modern medicine with those of related disciplines; (4) coordinating individual health behaviors and collective health actions (eg, community-based smoking cessation campaigns, vaccination programs, and health education activities); and (5) serving as the medical foundation of public health practice [1]. To maximize population health, it is crucial to identify conditions that should be prioritized for population-level management. Targeting the most prevalent, preventable, and impactful conditions enables more efficient allocation of resources and earlier intervention, leading to broad public health gains.

Noncommunicable diseases (NCDs) strongly linked to tobacco use are enumerated among the 7 NCDs and injury-related priority conditions that the Lancet Commission on Investing in Health identified as needing to be addressed to achieve its “50 by 50” goal of halving the probability of premature death by 2050 [2]. Tobacco-related NCDs include chronic obstructive pulmonary disease (COPD) and certain cancers, as well as deaths from atherosclerotic cardiovascular disease and hemorrhagic stroke [2]. In China, tobacco-related NCDs accounted for approximately 1 year of an estimated 3.5-year life expectancy gap attributable to the Lancet Commission’s 7 NCDs and injury-related priority conditions, relative to the North Atlantic regions. Furthermore, the portion attributable to tobacco-related NCDs represented approximately 23% of the overall 4.3-year life expectancy gap between the 2 regions [3]. The magnitude of this gap underscores the importance of managing tobacco-related NCDs at the population level, from preventive screening to disease management.

Among all tobacco-related NCDs, COPD is the most common respiratory disease. COPD is a leading cause of mortality and morbidity in China and worldwide [4,5]. It is the fourth leading cause of death and disability-adjusted life years among all diseases and was responsible for over 67.8 million deaths worldwide and approximately 11.7 million deaths in China in 2021 [6]. Recent estimates suggest that more than 400 million people are affected globally, with this number projected to reach 592 million by 2050 [7]. The immense health burden imposed by COPD also results in a substantial economic burden. It is estimated that COPD will cost China’s economy US $1.361 trillion (in 2017 international dollars) over the period 2020-50 [8], amounting to the largest economic burden from COPD faced by any country. The economic value of reducing avoidable mortality from COPD amounts to 1.4% of annual national income in 2019 and 0.9% in 2050; the 2019 figure places COPD as the fourth-ranked cause of death in terms of economic harm, as well as mortality, in China [9]. Moreover, COPD prevalence has steadily increased over the past 2 decades. Among the population aged 40 years and older, prevalence rose from 8% in 2002-04 to 14% in 2014-15, representing an increase of nearly 70% [10].

Two main challenges in COPD care have been widely discussed in the literature. First, awareness and early detection of COPD remain low among both the general and high-risk populations. Second, multimorbidity is frequently observed in patients with COPD and in those at high risk of developing the condition. Recent studies have shown an unmet health care need for COPD screening, formal diagnosis, treatment, and symptom control (ie, poor performance of the COPD care cascade) in China [11,12]. In a large-scale, population-based COPD screening and management program (the “Happy Breathing” program), it was estimated that among all patients with COPD who were spirometry-diagnosed within the program, 41.0% had previously undergone spirometry testing for the condition, 17.6% had been formally diagnosed with COPD, and 8.5% were currently receiving treatment. Among those receiving treatment, 4.6% had mild or no exacerbations in the prior year, and 3.9% had experienced no exacerbations [12]. Given the severe unmet health care need for COPD, increased attention to population-based COPD screening and interventions is warranted.

Community-based screening and management in populations at high risk for COPD represent one potential strategy to enhance awareness and early detection. For example, the TargetCOPD trial showed that screening ever smokers aged 40-79 years can lead to a significant increase in the detection of new COPD cases relative to usual practice; moreover, this approach has proven cost-effective as an active case-finding strategy [13]. Another recent study modeled a community-based COPD screening scenario and demonstrated its cost-effectiveness using a 2-step method [14]. Such research provides a solid scientific basis for conducting population-based COPD screening and management among individuals at high risk for COPD.

Multimorbidity among patients with COPD is extremely common, as these individuals often face behavioral risk factors, such as smoking, and tend to be older (indeed, nearly 50% of all adults suffer from multimorbidity at baseline) [15]. The reported prevalences of other chronic conditions among patients with COPD are high: more than 50% have hypertension [16], roughly 10%-60% experience anxiety and depression [17], 20% have diabetes [18], about 30% are obese and about 17% are underweight [16], 19% have chronic heart failure [18,19], 20% have ischemic heart disease [18,20], and 5% have had a stroke [18]. The high prevalence of coexisting conditions presents significant challenges for health care delivery, particularly with respect to designing care models that address multiple conditions simultaneously. A key challenge in delivering effective care to this population lies in how health care providers are incentivized. In many settings, clinician incentives remain aligned with passive diagnosis and treatment of individual diseases, rather than with providing proactive, inclusive care that holistically addresses multimorbidity.

In response to the substantial burden of COPD and associated multimorbidity, a comprehensive, population-based integrated model of screening and long-term care has been launched. However, there has been limited prior research evaluating the effectiveness of integrated management interventions within a population medicine framework that emphasizes proactive case identification and management among high-risk groups, rather than relying solely on reactive clinical care. Therefore, an important goal of this study is to evaluate the effectiveness of the newly launched multimorbidity care model in addressing the growing challenges of COPD and multimorbidity. In this protocol, we implement an integrated multimorbidity intervention package to evaluate its impact on relevant health outcomes among individuals diagnosed with COPD or at high risk of COPD. We also introduce a performance-linked “pay-for-population” scheme that ties financial incentives for health care workers to concrete population-level indicators, such as spirometry coverage, diagnosis rate, treatment initiation rate, and reduction in acute exacerbations. The design of the integrated package and the decision to evaluate its effects as a whole are based on consensus with the local authority to ensure feasibility in a real-world setting. This combined strategy is intended to shift provider behavior from passive disease response to proactive, integrated care delivery in a manner aligned with the principles of population medicine. This study is part of the Population Medicine Multimorbidity Intervention in Xishui County (POPMIX) project, which aims to develop and evaluate evidence-based, multicomponent strategies for managing chronic diseases and their comorbidities in a resource-limited rural county in China. We aim to investigate whether a population-based integrated multimorbidity intervention package affects our primary outcomes, including the number of chronic diseases controlled, whether participants have received lung function testing, and forced expiratory volume in 1 second (FEV_1_), as well as our secondary outcomes, such as health-related quality of life, mental health, behavioral risk factors, health care utilization, productivity loss, knowledge of COPD and asthma, and care cascade indicators for COPD and other chronic diseases.

## Methods

### Trial Design

**Figure 1:**
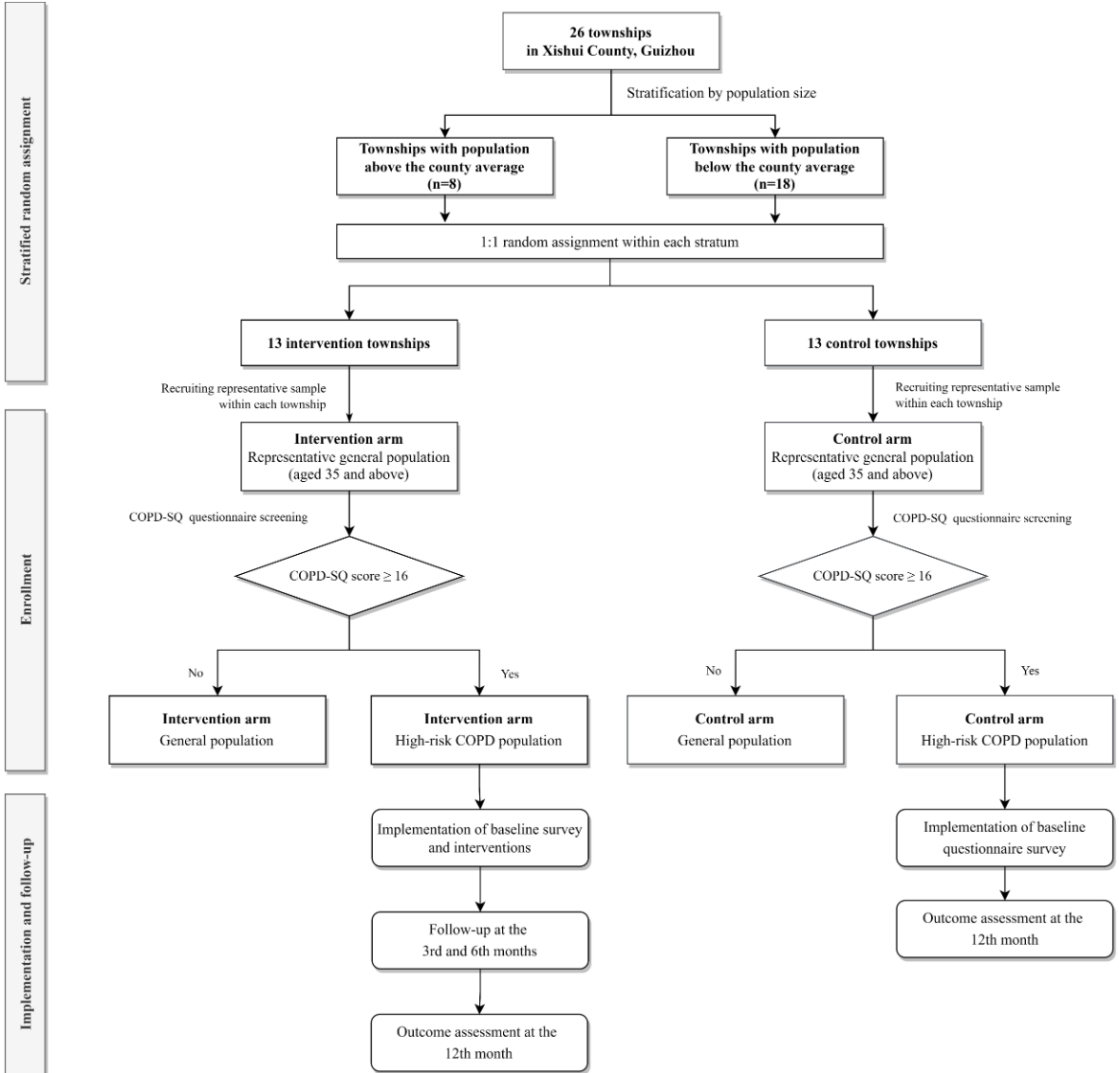
Trial flowchart. COPD: chronic obstructive pulmonary disease; COPD-SQ: COPD Screening Questionnaire. See Multimedia Appendix 4 for a higher resolution version of this figure.

This is a parallel, 2-arm, stratified cluster randomized controlled trial (cRCT) conducted in Xishui, Guizhou, China (Figure 1). The protocol was designed in accordance with the Standard Protocol Items: Recommendations for Interventional Trials (SPIRIT) 2025 Statement. The SPIRIT checklist for this protocol is provided in Multimedia Appendix 1.

Our clusters are the townships of Xishui County. The demographic characteristics of Xishui County, including the number and size of townships, are summarized in Table 1. The townships were stratified according to whether their population size exceeded the mean size of all townships. Within each stratum, townships were randomly allocated in a 1:1 ratio to either the intervention or control arm using a computer-generated randomization sequence. Participant recruitment was based on a comprehensive roster of permanent residents aged 35 years and above, provided by the local government as of May 10, 2024, with individuals selected from each township for enrollment. Permanent residents were defined as individuals who had resided in Xishui County for at least 3 months and were expected to remain within the same township for the subsequent year at the time the government collected the information.

**Table 1:** Characteristics of Xishui County, Guizhou.

Characteristics	Xishui County
Total population size (10,000^a^), n	30.20^a^
Rural townships, n	22
Villages within rural townships, n	210
Average rural township size (ie, number of people), n	9518
Urban townships, n	4
Communities within urban townships, n	47
Average urban township size (ie, number of people), n	23,150

^a^The total population size listed in Table 1 was obtained from the Xishui authority. The figures here reflect permanent residents as of May 10, 2024, and are limited to individuals who had already stayed in Xishui County for 3 months in 2024 and would continue to stay within the same township for the next year.

The COPD Screening Questionnaire (COPD-SQ) was used to identify individuals at high risk for COPD. The COPD-SQ includes 7 items: age, cumulative smoking history, BMI, cough, breathlessness, family history of respiratory diseases, and exposure to cooking-related smoke [21]. The total score ranges from 0 to 38, with higher scores indicating a greater risk of developing COPD. This questionnaire has been validated through various epidemiological surveys and community health screenings and has been recommended for COPD screening in primary care settings in China [21]. Participants with a COPD-SQ score of 16 or higher were considered for inclusion in the study, ensuring that the cohort comprised a population at high risk for COPD.

### Setting

This study was implemented across 26 township-level clusters in Xishui County, a mountainous area in northern Guizhou Province, China, where the prevalence of smoking was 37.9% in 2018-2019, the second highest among all provinces [22]. A rural area designated as China’s National Comprehensive Primary Health Experimental Area, Xishui presents a unique combination of policy-driven innovation and socioeconomic challenges. With a gross domestic product per capita (48%) below the national average in 2024 and limited human capital development, the county exemplifies a resource-constrained rural setting. These conditions, combined with its experimental role in health system transformation, have established Xishui as an ideal setting for evaluating scalable multimorbidity interventions tailored to underserved populations.

### Trial Participants (Inclusion and Exclusion Criteria)

All research participants were required to be at least 35 years old. In addition, eligible participants had to meet the following criteria: (1) a COPD-SQ score of 16 or higher, corresponding to individuals at high risk for COPD; (2) residence in a township of Xishui County for the prior 3 months and plans to remain in the same township for the upcoming year; and (3) provision of informed consent.

Individuals with severe cognitive disorders that significantly impair comprehension, decision-making, or adherence to the intervention were excluded. Similarly, those who had completely lost the ability to perform activities of daily living independently were not eligible for participation. These criteria ensure that the study includes individuals who can actively engage in the intervention and follow-up assessments while minimizing confounding variables.

### Research Ethics Approval

This study received ethical approval from the Ethics Committee of Peking Union Medical College. To protect participant privacy, all identifying information has been removed from the dataset, and data are anonymized before analysis. Participants have the right to withdraw from the study at any point, without consequences for their standard health care access. The study is committed to upholding the highest standards of ethical research conduct, ensuring that all participants are treated respectfully and that their health and personal information are safeguarded throughout the research process. This study was initially approved by the Ethics Committee of Peking Union Medical College (approval number CAMS&PUMC-IEC-2024-040). A continuing ethics review was completed in June 2025, and updated approval was granted under approval number CAMS&PUMC-IEC-2025-061.

### Intervention

Participants assigned to the intervention arm received access to the population medicine multicomponent intervention package, which was developed through iterative prototyping and stakeholder consultations. The specific eligibility criteria for each intervention are summarized in Table 2. Participants in the control arm were notified of their COPD risk status and encouraged to complete a face-to-face interview; they were not provided with any interventions after the interview, but were encouraged to receive usual care.

**Table 2:** Eligibility criteria for interventions.

Population level and target population		Intervention	Eligibility criteria
**General population**			
	General population	Health education	Permanent residents
	General population	Online screening with the COPD^a^-SQ	Permanent residents aged 35 years and above
**Patients with COPD or high-COPD-risk population**			
	High-COPD-risk population	Community-based spirometry pulmonary function tests, interpretation of results, and health education	Individuals with COPD-SQ^b^ score ≥16
	Smokers within high-COPD-risk population	Health education for smokers to support smoking cessation	Currently smoking or have quit within the last 6 months
	Smokers within high-COPD-risk population	Digital health interventions for smoking cessation	Currently smoking or have quit within the last 6 months and own a smartphone
	High-COPD-risk population with depression and anxiety symptoms	Mental health digital health interventions	Individuals with Warwick Edinburgh Mental Well-being Scale score <45 who own a smartphone
	High-COPD-risk population with mental health symptoms	Health education for smokers with mental health issues	Individuals with Warwick Edinburgh Mental Well-being Scale score <45
	High-COPD-risk population with abnormal weight	Weight abnormality interventions	Individuals with BMI <18.5 kg/m^2^ or BMI ≥ 24.0 kg/m^2^
	High-COPD-risk population with hypertension	Hypertension management and education	Three consecutive measurements with average systolic blood pressure ≥140 mmHg or diastolic blood pressure ≥90 mmHg or both
	High-COPD-risk population with type 2 diabetes mellitus	Diabetes management and education	Fasting blood glucose ≥7.0 mmol/L or random blood glucose ≥11.1 mmol/L
	Patients with COPD or high-COPD-risk population who fail to conduct pulmonary function tests	Encouragement to conduct computed tomography scan and seek professional medical treatment	Individuals with FEV_1_^c^/FVC^d^ <0.7 after bronchodilation, or high-COPD-risk population who fail to conduct a pulmonary function test for any reason
**Health providers**			
	Health providers	Intrinsic incentive mechanism	Primary care providers who engage with the investigation and intervention
	Health providers	Extrinsic incentive mechanism	Primary care providers who engage with the investigation and intervention

^a^COPD: chronic obstructive pulmonary disease.

^b^COPD-SQ: COPD Screening Questionnaire.

^c^FEV_1_: forced expiratory volume in 1 second.

^d^FVC: forced vital capacity.

**Figure 2:**
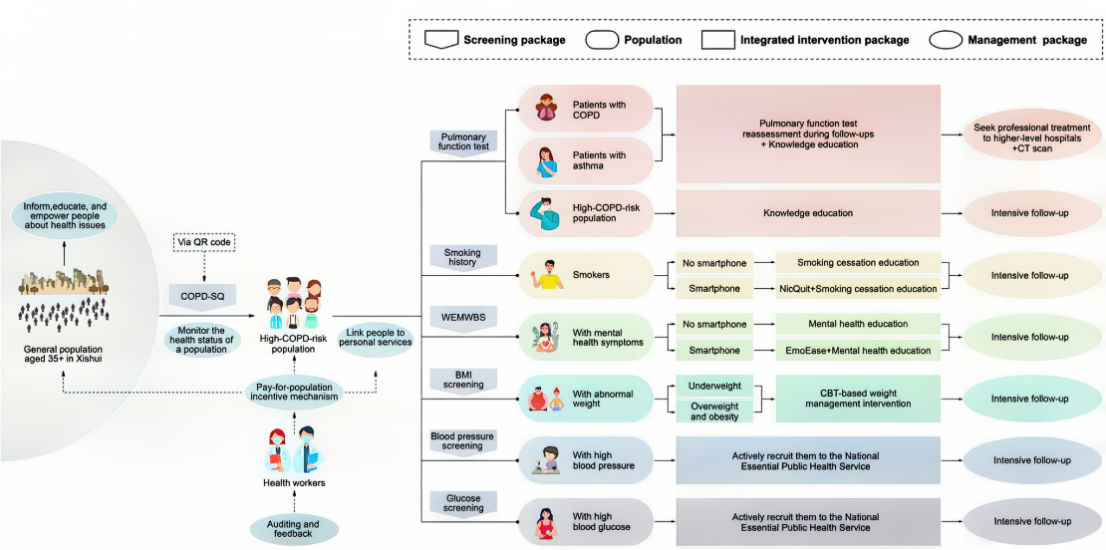
Integrated pathway of the multicomponent intervention package. CBT: cognitive behavioral therapy; COPD: chronic obstructive pulmonary disease; COPD-SQ: COPD Screening Questionnaire; CT: computed tomography; WEMWBS: Warwick-Edinburgh Mental Well-Being Scale. See Multimedia Appendix 4 for a higher resolution version of this figure.

The strategies included in the multicomponent intervention package within this trial have been proven cost-effective and are advocated by the Lancet Commission on Investing in Health [2]. Figure 2 illustrates the full integration pathway of the intervention package implemented in this trial. The structure comprises 4 layers: the screening package, the targeted population, the integrated intervention components, and the long-term management package. The figure shows how the general population is first screened and stratified into risk groups, followed by targeted interventions (eg, spirometry, mental health support, and smoking cessation) and intensive follow-up. It visualizes the conceptual transition from individual treatment to proactive population health management.

**Figure 3:**
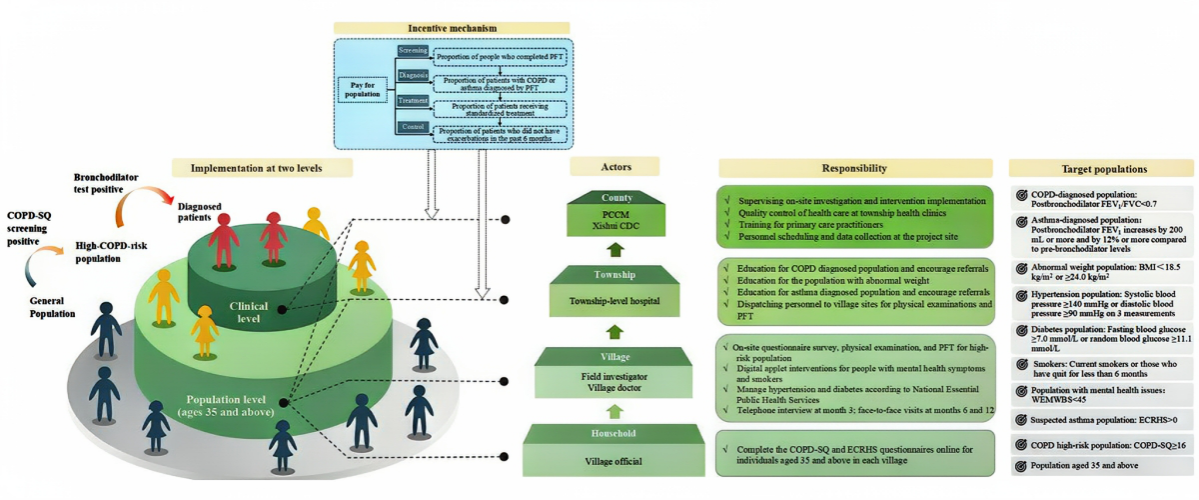
Implementation structure and execution mechanism of the multicomponent intervention package. CDC: Centers for Disease Control and Prevention; COPD: chronic obstructive pulmonary disease; COPD-SQ: COPD Screening Questionnaire; ECRHS: European Community Respiratory Health Survey; FEV1: forced expiratory volume in 1 second; FVC: forced vital capacity; PCCM: pulmonary and critical care medicine; PFT: pulmonary function test; WEMWBS: Warwick-Edinburgh Mental Well-Being Scale. See Multimedia Appendix 4 for a higher resolution version of this figure.

In addition to the intervention components and care pathways described in Figure 2, the implementation of the multicomponent intervention package was supported by a multilevel delivery structure and a performance-linked incentive mechanism. Figure 3 illustrates the organizational structure, population stratification, incentive design, and distribution of responsibilities across administrative levels (county, township, village, household) for delivering the intervention in Xishui. The figure also specifies the eligibility criteria for each target subpopulation, clarifying how risk stratification and service delivery were operationalized in the field.

### Specific Interventions

#### Overview

Specific interventions as part of the multicomponent intervention package are described in the following sections.

#### Health Education

Health education is provided to all permanent residents, regardless of their risk level. This intervention aims to increase population-wide awareness of tobacco-related NCDs, particularly COPD, and to promote early health-seeking behaviors. Health education is delivered through multiple channels, including face-to-face sessions led by trained primary care workers, printed brochures, posters in public areas, and short videos/messages distributed via WeChat (Tencent Holdings Limited). Educational content covers the importance of lung health, risk factors such as smoking and indoor air pollution, the purpose of spirometry screening, and strategies for chronic disease prevention and lifestyle modification.

#### Online Screening for COPD

As part of the screening package, all representative permanent residents aged 35 years or older are invited to complete an online screening survey via a QR code–linked digital platform. The survey incorporates the COPD-SQ and is designed to allow broad dissemination and efficient data collection. Upon submission, participants receive automated feedback on their risk level and are guided to appropriate follow-up actions, such as spirometry appointments or health counseling sessions.

#### Community-Based Spirometry Pulmonary Function Tests, Results Interpretation, and Health Education

The population at high risk of COPD in the intervention arm receives alerts directing them to a community gathering place for spirometry testing, which is conducted using the BH-AX-MAPG spirometry equipment (BreathHome). Participants screened for COPD are referred to the county hospital for computed tomography and formal diagnosis. In addition, they receive further health education on the risks of COPD and strategies for disease prevention and management, delivered verbally by primary health care providers and supplemented with printed materials for distribution.

#### Health Education for Smokers to Support Smoking Cessation

Participants in the intervention group receive targeted health education to reinforce the importance of smoking cessation. This education emphasizes the health risks associated with smoking and the benefits of quitting, providing participants with evidence-based information and practical guidance. It is delivered through verbal communication by primary health care providers and supplemented with printed posters for distribution.

#### Digital Health Interventions for Smoking Cessation

NicQuit is a WeChat-based digital smoking cessation intervention that includes cognitive behavioral therapy (CBT) modules focused on smoking cessation strategies, coping with triggers, and reinforcement techniques to maintain abstinence. It is designed for smokers who are currently smoking or have quit within the past 6 months [23]. The intervention specifically targets individuals familiar with smartphone technology, ensuring accessibility and usability. Personalized notifications and reminders are delivered through the WeChat platform to encourage participants to engage regularly with the cessation plan and maintain adherence.

#### Mental Health Digital Health Interventions

A CBT-based digital mental health intervention, EmoEase [24], is offered to individuals experiencing mental health symptoms (Warwick-Edinburgh Mental Well-Being Scale [WEMWBS] < 45) who have a smartphone. This WeChat-based program includes psychoeducation, mood tracking, guided CBT exercises, and self-regulation techniques. The program emphasizes the link between mental health and respiratory symptoms, aiming to enhance coping, treatment adherence, and sustained behavior change.

#### Health Education for High-COPD-Risk Individuals With Mental Health Symptoms

Specialized health education is offered to high-COPD-risk individuals with coexisting mental health symptoms, tailored to their specific challenges. This education includes guidance on managing mental health symptoms and is delivered verbally by trained community health workers or general practitioners, supplemented with printed materials for distribution.

#### Weight Abnormality Interventions

Individuals with a BMI<18.5 kg/m^2^ (underweight) or ≥24.0 kg/m^2^ (overweight or obese) are considered to have weight abnormalities. CBT-based motivational interviewing is used to help participants self-identify weight-related barriers before providing tailored health education. During the intervention session, participants are asked CBT-informed questions designed to prompt reflection on the disadvantages and advantages of being underweight or overweight.

#### Hypertension and Diabetes Management

The goal of this intervention is to actively enroll smokers whose blood pressure exceeds 140/90 mmHg [25] or whose random blood glucose exceeds 11.1 mmol/L (or fasting blood glucose ≥ 7.0 mmol/L) into the National Essential Public Health Service in China [26]. These participants also receive health education on hypertension and diabetes through verbal counseling by trained community health workers or general practitioners, supplemented with printed materials for distribution.

#### Encouragement to Seek Professional Medical Treatment in Superior Hospitals

Patients with a postbronchodilator FEV_1_-to-forced vital capacity (FVC) ratio of <0.7 are diagnosed with COPD and are encouraged to seek professional medical care at higher-level hospitals for further diagnosis and management. Some participants at high risk of COPD failed to complete pulmonary function testing due to contraindications, lack of understanding of the procedure, failure to meet spirometry quality-control standards, or unwillingness to undergo the tests. High-risk participants who do not complete pulmonary function testing for any reason are encouraged to undergo a computed tomography scan and seek formal diagnosis and medical treatment at a superior hospital.

#### Pay-for-Population Mechanism

A novel pay-for-population mechanism is introduced to motivate primary care providers to actively engage with the intervention by aligning compensation with population health goals. This approach ties provider payment to 4 key stages of care: screening, diagnosis, treatment, and control. Health providers in township hospitals are rewarded based on 4 performance indicators. The first is the proportion of individuals completing pulmonary function testing among township residents aged 35 years and above. The second is the proportion of individuals diagnosed with COPD among those identified as high risk in the initial screening (defined as COPD-SQ >16). The third is the proportion of patients with confirmed COPD who receive standardized inhaled treatment, relative to the total number of patients with confirmed COPD in the township. The fourth is the proportion of patients with confirmed COPD who have not experienced an acute exacerbation in the past 6 months, relative to all patients with confirmed COPD in the township. The county hospital’s respiratory department and the county Center for Disease Control and Prevention are assessed using the same 4 performance indicators, with data aggregated across all 13 intervention townships. In addition to providing an extrinsic, results-based financial incentive, providers are offered specialized training and capacity-building opportunities to appeal to intrinsic motivation, support proactive service delivery, and foster a strong sense of responsibility for population health. Details on implementing the pay-for-population mechanism are provided in Multimedia Appendix 2.

### Outcomes

We will measure both primary and secondary end points at the end of follow-up for both trial cohorts. End points will be assessed among individuals assigned to the intervention group and compared with those among individuals assigned to the control group (ie, intent-to-treat). This high-COPD-risk population cohort will be followed longitudinally through March 2026 for repeated measurement of end points and covariates, according to the initial intervention versus control assignment. Specifically, all primary and secondary end points will be measured during health worker visits at 6 and 12 months after assignment, except for knowledge of diseases, health service utilization, and the number of chronic diseases controlled, which will be assessed only at the end of the trial (ie, 12 months after assignment). Table 3 defines the trial’s primary outcomes, illustrating the functional forms of measured variables and the approach to assessing each end point, while secondary outcomes are detailed in Multimedia Appendix 3.

**Table 3:** Primary outcomes.

Outcome	Description
Number of chronic conditions controlled	Definition: Number of controlled conditions among 7 objectively measured chronic health conditions: chronic obstructive pulmonary disease, asthma, depressive symptoms, anxiety symptoms, BMI, hypertension, and type 2 diabetes. Functional form: Counting data Measurement: Through objective physical examination or a validated scale
COPD^a^ screening	Definition: Response to the question “Have you ever had a pulmonary function test?” Functional form: Binary Measurement: Self-reported response
FEV_1_^b^ measurement	Definition: Volume of air forcibly exhaled during the first second of a forced expiratory maneuver Functional form: Continuous Measurement: Pulmonary function test and portable spirometry

^a^COPD: chronic obstructive pulmonary disease.

^b^FEV_1_: forced expiratory volume in 1 second.

### Timeline

Our trial began on June 17, 2024, with recruitment conducted across all townships in Xishui County. Participants in the intervention arm undergo structured assessments at 4 time points: baseline (in-person), 3-month follow-up (telephone), 6-month follow-up (in-person), and 12-month follow-up (in-person). At baseline, following completion of informed consent, participants were invited to undertake a comprehensive in-person assessment. Physical examinations and pulmonary function tests are conducted at multiple points, with protocols tailored by subgroup: prebronchodilator spirometry at baseline and 12 months for the high-COPD-risk population; postbronchodilator spirometry at baseline and 12 months for patients with COPD; and prebronchodilator spirometry at 6 months for high-COPD-risk participants with prebronchodilator FEV_1_/FVC<70% at baseline. All baseline assessments were administered under the supervision of trained site staff to ensure accuracy and adherence to protocol standards. Subsequent follow-ups include a 3-month telephone assessment to monitor intervention adherence and health status. In-person follow-ups at 6 and 12 months repeat elements of the baseline assessment, including the questionnaire, physical examination, and pulmonary function testing. For the control group, the schedule includes a baseline questionnaire and a 12-month on-site survey. Table S2 in Multimedia Appendix 3 presents the timeline of prospective cohort visits and data collection.

### Sample Size

We included all 26 townships in Xishui County and allocated them to the intervention or control group using 1:1 stratified randomization. Based on the population size of each township, we recruited a representative sample of 44,000 residents aged 35 years or older. All 44,000 participants underwent COPD-SQ screening via QR code. Considering a 10% attrition rate and an estimated high-risk prevalence of approximately 25% among individuals aged 35 years and above [27], the projected final enrollment is 10,000 individuals in the high-COPD-risk population.

Because of the relatively large number of important secondary end points and the high degree of uncertainty regarding the prevalence and risk factors of COPD in rural and urban communities, we chose FEV_1_ as one of the primary end points of interest and conducted the power calculation conservatively. This choice was made because FEV_1_ is an objective and clinically relevant measure that helps address uncertainties in previous studies while allowing for a more robust power calculation. Given that 3 outcomes are designated as primary end points, we applied a Bonferroni adjustment for both sample size and power calculations. According to the China Pulmonary Health Study—a cross-sectional study in a nationally representative sample of adults aged 20 years or older—the overall prevalence of spirometry-defined COPD is expected to be 8.6% (95% CI 7.5%-9.9%) [10]. Based on a study conducted by Chen et al [28], the mean and SD of FEV_1_ levels among patients with mild COPD and the high-COPD-risk population in China were reported to be 2.62 L and 0.55 L, respectively. Following standard procedure, the intraclass correlation coefficient (ICC) was identified in advance, as it is a key parameter required for accurate sample size estimation in cluster randomized designs. Previous literature reports an ICC of 0.055 for patients with COPD in the PACK Brazil Study and 0.05 in the Danish TeleCare North Study [29]. Thus, we set the ICC at 0.05 for sample size calculation. We followed the formula for population-based stratified cRCTs proposed by Crespi [30] and assumed a 2-sided significance level of 1.67% under the Bonferroni adjustment and a power of 80%. Based on these assumptions, we calculated a conservative sample size estimate of 2850 individuals in the high-COPD-risk population, which is required to detect a minimal difference of 0.1 L in FEV_1_ in our study.

### Power Calculations

In our power calculations, we estimated the minimum detectable differences (MDDs) in FEV_1_ (L) levels under varying sample sizes and ICC values, assuming a 2-sided significance level of 5% and a power of 80%. We further assumed that 10% of participants would refuse to participate in the trial and that 10% of individuals could not be followed up for end point assessment. To our knowledge, this is the first real-world, large-scale multimorbidity implementation trial, and no similar trials are available for reference. Therefore, we conducted a sensitivity analysis to examine how the MDDs in FEV_1_ levels change with different proposed population sizes. The results of this sensitivity analysis are presented in Table 4. Our analyses indicate that the population-based cluster randomized trial design can detect differences of 0.052-0.218 L in FEV_1_ levels between the treatment and control groups. In the implementation, we applied stratification before randomization to reduce between-cluster variance.

**Table 4:** Minimum detectable differences in forced expiratory volume in 1 second (liters) across ICC^a^ values and proposed sample sizes.

Proposed sample size, n	ICC=0.005	ICC=0.01	ICC=0.025	ICC=0.05
5000	0.089	0.113	0.165	0.226
5500	0.085	0.108	0.157	0.216
6000	0.081	0.103	0.151	0.207
7000	0.075	0.095	0.139	0.191

^a^ICC: intraclass correlation coefficient.

As shown in Table S2 in Multimedia Appendix 3, we listed 3 primary outcomes and a large set of secondary outcomes. For primary outcomes, we will apply the Bonferroni adjustment, setting the significance threshold at 0.0167 (0.05/3). For secondary outcomes, to mitigate concerns of type I error inflation, we plan to use the Benjamini-Hochberg procedure to control the false discovery rate. We will set the false discovery rate at 0.05 and rank all unadjusted *P* values of the secondary outcomes from smallest to largest. Adjusted *P* values will then be reported following the Benjamini-Hochberg procedure, and the significance of each secondary outcome will be determined based on these adjusted *P* values.

In Table 5, we present a sensitivity analysis reporting Cohen *d* for the trial. We set the SD to 1 to explore Cohen *d* (standardized MDDs) under different ICC values and proposed sample sizes.

**Table 5:** Minimum detectable differences in standardized mean difference (Cohen d) across ICC^a^ values and proposed sample sizes.b

Proposed sample size, n	ICC=0.005	ICC=0.01	ICC=0.025	ICC=0.05
5000	0.162	0.205	0.300	0.412
5500	0.155	0.196	0.286	0.393
6000	0.148	0.188	0.274	0.376
7000	0.137	0.174	0.253	0.348

^a^ICC: intraclass correlation coefficient.

^b^Minimum detectable difference values represent standardized mean differences (Cohen *d*), calculated by assuming a pooled SD (σ) of 1 for the outcome.

### Assignment of the Intervention

This cRCT assigned participants to either the population medicine multicomponent intervention or the control arm at the township level, with randomization stratified by geographic region to ensure balance across community characteristics. A computer-generated algorithm was used to randomly allocate 13 townships to each arm, with the sequence generated by an independent statistician who was not involved in recruitment or intervention delivery. Given the nature of the intervention, this is an open-label trial, meaning health care providers and research staff are aware of group assignments. However, participants themselves were not explicitly informed of their allocation or that they were part of an “intervention group,” to mitigate expectation bias. No additional interventions were provided to the control group, although usual care remained accessible throughout the study period.

To enhance adherence to the intervention protocol, a structured approach was integrated into all follow-up phases. Adherence was reinforced through telephone consultations at the 3-month follow-up and through an in-person visit at the 6-month follow-up. During these sessions, participants received personalized feedback on their health outcomes (eg, spirometry results, smoking status), including comparisons with baseline data to highlight improvements or areas requiring attention. For example, if a participant had not sought professional medical treatment for COPD, a root-cause analysis was conducted by reviewing baseline results and exploring potential barriers, such as lack of motivation or difficulty accessing health care. Adherence was systematically monitored at each follow-up through validated questionnaires assessing compliance with the intervention (eg, whether the participant had sought care, app usage frequency, smoking cessation attempts). In addition, automated reminders and tailored counseling were provided to address gaps in adherence. This iterative process ensured that each follow-up served as an opportunity to motivate participants, troubleshoot challenges, and adapt strategies to sustain engagement throughout the trial.

### Blindness

This is an open-label cRCT, in which field workers and participants are not blinded. We consider this scientifically reasonable for large-scale population health intervention trials conducted in real-world community settings, such as the POPMIX trial. However, we made efforts to blind most members of the Data and Safety Monitoring Board (DSMB). Statisticians will remain blinded when conducting data analyses, and DSMB members will not be informed of the exact intervention assignment of each township. Thus, while full blinding was not feasible, we maintained investigator blinding wherever operationally possible, consistent with best practices in open-label population health and behavioral intervention trials.

### Data Collection Plan

We are collecting trial data across multiple dimensions to comprehensively assess the impact of the multicomponent intervention (Table 2). At baseline, all participants completed a structured questionnaire capturing demographics and socioeconomic status, quality of life, lifestyle and risk behaviors, disease history, mental health status (9-item Patient Health Questionnaire [PHQ-9], 7-item Generalized Anxiety Disorder [GAD-7], and WEMWBS), and productivity loss. In addition to self-reported data, participants underwent physical examinations, including measurements of height, weight, BMI, waist circumference, heart rate, blood pressure, and blood glucose. Pulmonary function was assessed using spirometry to evaluate respiratory health and detect airflow limitation. For all participants in the high-COPD-risk population, prebronchodilator spirometry was conducted at baseline and again at 12 months to monitor changes in lung function over time. For participants diagnosed with COPD, both pre- and postbronchodilator spirometry were performed at baseline, with prebronchodilator tests repeated at 6 months and both pre- and postbronchodilator tests repeated at 12 months.

Follow-up data are collected at 3 time points: 3 months (via telephone), 6 months (in-person), and 12 months (in-person) for the intervention group and only 12 months for the control group (in-person). At each in-person follow-up, participants complete key sections of the baseline assessments, including self-reported data on COPD symptoms and management (lung function via spirometry, exacerbations, treatment adherence, COPD Assessment Test, and Modified Medical Research Council), asthma symptoms and management, high-risk behaviors (eg, daily cigarette consumption and smoking dependence, alcohol use), and chronic disease status (blood pressure, blood glucose, and BMI). Mental health status is reassessed using the same scales (PHQ-9, GAD-7, and WEMWBS), and health-related quality of life is measured using the EQ-5D-5L and the Saint George Respiratory Questionnaire for patients with COPD. Additionally, lifestyle behaviors, such as physical activity, alcohol consumption, and diet (including sugar, salted vegetable, and general vegetable intake), are monitored through self-report. Health resource utilization, including outpatient and inpatient visits, is also recorded. Data are collected by trained field staff using standardized protocols to ensure accuracy and reliability. All data are entered electronically via an electronic data capture (EDC) system, which includes built-in validation checks to minimize data entry errors.

### Data Management

This study uses an EDC system to ensure real-time data entry, automated validation, and secure storage. Data are collected by trained field staff using tablet-based digital surveys that sync directly with a central database. The EDC system incorporates range checks, logical consistency validations, and missing data alerts to minimize errors, with flagged inconsistencies promptly reviewed by the data management team. Data collection forms, covering demographic, clinical, and behavioral assessments, are standardized and precoded, while biometric data (eg, pulmonary function, blood pressure, BMI, and blood glucose) are recorded directly into the system. Spirometry data are transmitted from portable devices and subjected to automated quality control checks to ensure validity.

We store data using numeric identifier codes to maintain participant anonymity in a password-protected database accessible only to the research team for analysis. Access is restricted to a minimal number of individuals, including the principal investigator, co-investigators, biostatisticians, and data scientists.

### Statistical Analysis

Primary analyses will compare outcomes between baseline and the 12-month follow-up using generalized linear mixed models (GLMMs) with township as a random effect under the intention-to-treat principle. To evaluate the robustness of the findings, intervention effects will be estimated using a primary design-adjusted model, which will adjust for stratification factors and baseline values of the outcome measure. This will be complemented by a sensitivity-adjusted model that will further incorporate demographic covariates, such as age, sex, and education level. Adjustment for multiple testing will be applied using the Bonferroni correction and Benjamini-Hochberg procedure.

To evaluate the effects of individual intervention components and explore which components drive specific outcomes, we designed a series of regression discontinuity design analyses, planned for publication after the main trial results. We established 7 clear diagnostic thresholds for the conditions included in our trial. For COPD, the threshold is an FEV_1_-to-FVC ratio of 70%. For PHQ-9 and GAD-7, a score of 10 is used to indicate the presence of depressive and anxiety symptoms, respectively. For the WEMWBS questionnaire, a threshold of 45 is used to indicate whether general mental health is adequate. For hypertension, clear thresholds are defined as 140 mmHg for systolic blood pressure or 90 mmHg for diastolic blood pressure. Individuals with a BMI below 18.5 kg/m^2^ are considered underweight, while those with a BMI above 24 kg/m^2^ are considered overweight. For high blood glucose, thresholds of 7.0 mmol/L for fasting glucose or 11.1 mmol/L for random glucose indicate potential type 2 diabetes mellitus. These well-defined thresholds allow us to conduct regression discontinuity design analyses to evaluate the component-specific effects of the integrated intervention package within the intervention arm.

To better elucidate how the intervention package will reshape the behaviors of both high-COPD-risk populations and health care providers, we also designed a series of qualitative studies involving interviews with high-COPD-risk populations, physicians and community health workers, and policy makers in the intervention arm. These studies will serve as important supplements to the main trial by illustrating the mechanisms of the intervention and the subjective experiences of being intervened under the population medicine framework.

### Monitoring

We appointed an independent DSMB to provide scientific rigor and ensure an independent evaluation of safety. The DSMB comprised leading experts in public health, epidemiology, clinical research, and respiratory disease. The DSMB convened 3 meetings in which baseline and follow-up descriptive analyses for the intervention arm were evaluated for process monitoring. The DSMB convened for a formal interim analysis in February 2026. At this meeting, the research team reviewed the conduct of the trial, including adherence to intervention components, logistical issues, and the completeness and quality of the collected data. Our biostatisticians also presented emerging effect estimates for the intervention and control arms to the steering committee. Stopping rules were established regarding safety and study completion:

- If deaths or severe adverse effects were reported, these would be immediately conveyed to the DSMB. The DSMB would determine if the deaths or adverse effects were related to the intervention and advise if the study should be stopped immediately.
- In February 2026, during the planned interim analysis, the trial was approaching its completion as field workers confirmed that the majority of eligible participants had been contacted. At this stage, the results were assessed to determine the study’s conclusion based on the following:
  - Null findings, suggesting that the intervention had no detectable effect and indicating that the study should be stopped.
  - Statistically significant findings, indicating that the study had achieved its primary objectives and should be stopped.
  - Emerging but statistically insignificant effects, suggesting that the study might require further observation if applicable. However, the study would be terminated if field workers confirmed that all eligible participants had been contacted and no additional individuals remained to undergo follow-up assessments.

### Plans for Communicating Important Protocol Amendments to Relevant Parties

The Ethics Commission of the Medical Faculty of Peking Union Medical College was contacted and notified of any protocol amendments requiring its approval. Following approval by the Ethics Committee and before implementation, all research team members were informed of the protocol amendments.

In this trial, an unavoidable amendment was made to the secondary outcome measures. Although outcome modifications are generally discouraged, this change was necessitated by a nonnegotiable regulatory restriction. Specifically, we initially planned to measure health care utilization (ie, outpatient visits, inpatient visits, and medical expenditure) using electronic health records from medical institutions in Xishui County. However, due to unexpected government regulations, we were unable to reach an agreement to obtain these data. This amendment was approved by the Ethics Committee, and all research personnel were notified before implementation to ensure consistency in data collection and analysis.

### Consent and Withdrawal

We provided written study information and the informed consent form to eligible individuals. The field workers explained the study’s aims and detailed procedures, in the presence of a witness if required. Participants were given sufficient time to decide whether to participate in the study and the opportunity to ask questions, which were answered accordingly. For illiterate participants, consent was documented before enrollment using a thumbprint signature and a witness’s signature.

We informed eligible participants about the risks and benefits of participation in the trial. A decision not to participate in the study did not carry any consequences for the individual. All participants were free to refuse or discontinue data collection at any stage, and participation did not require relinquishing any concomitant care. Although there were no predefined criteria for modifying or discontinuing allocated interventions, reasons for attrition will be documented and reported in future publications.

### Confidentiality

Throughout the study, all data will be handled with strict confidentiality, and data collection will conform to the requirements of national data protection legislation. Digital data will be stored in password-protected files at the Center of the Chinese Academy of Medical Sciences and will be accessible exclusively to research team members. Third parties may receive anonymized data solely for research purposes. Informed consent forms, laboratory books, and other participant-related documents will be securely stored during the conduct of the study and will subsequently be stored at the co-principal investigators’ (CW, SC, and TY) office premises.

### Dissemination

Upon completion of the trial, participant-related information and results will be provided to the participants themselves or to their families. The trial has gained public attention since it was first introduced in the Lancet Commission on Investing in Health and now serves as a critical component of the ongoing Lancet Commission Report on Population Medicine and Public Health. We will disseminate the trial results through international peer-reviewed journals and at other renowned international academic conferences.

## Results

Data collection for the POPMIX-COPD trial began in June 2024. Baseline, 3-month, and 6-month assessments have been completed. The trial is planned to finish data collection by March 2026. Data analyses will be conducted after all data have been collected and the dataset has been locked. We plan to publish the POPMIX-COPD paper before mid-2027. At the time of protocol submission, the trial status is active. Recruitment began on June 17, 2024, and was completed on December 31, 2024. Follow-up data collection will conclude on March 31, 2026.

## Discussion

To the best of our knowledge, this is the world’s first large-scale, community-based population medicine multimorbidity intervention study targeting tobacco-related NCDs among a high-COPD-risk population. As The Lancet Commission on Investing in Health has suggested, tobacco use–related NCDs are among the leading modifiable risk factors contributing to life expectancy gaps in China. However, awareness of tobacco-related NCDs remains low; China is the world’s largest producer and consumer of tobacco, and the health and economic burdens of tobacco-related NCDs are substantial, especially in rural China [31]. Against this context, our population medicine trial is designed to improve population health and maximize collective welfare. Through this study, we aim to evaluate whether an integrated multimorbidity intervention based on population medicine theory can enhance health outcomes and awareness of tobacco-related NCDs in a relatively low-resource setting such as Xishui County.

Our population medicine trial emphasizes population health rather than individual outcomes. Unlike most clinical trials that predominantly focus on patients actively seeking care within the health system, our study targets high-COPD-risk individuals in the general population and applies community-level interventions through the use of the COPD-SQ. Early detection and intervention to prevent disease progression among the high-COPD-risk population, including individuals with undiagnosed conditions or unmet care needs, are essential for improving population health.

We also extended the scope of our study to population health outcomes, such as multimorbidity. We specified the “number of chronic diseases controlled” as a primary outcome to capture the intervention’s effects not only on COPD but also on common co-occurring conditions, such as hypertension, diabetes, smoking dependence, abnormal BMI, asthma, and symptoms of depression and anxiety. Given that our target population is a high-COPD-risk population, we considered measurement of FEV_1_ to be another essential primary outcome. Finally, we included whether participants had ever received lung function testing as a primary outcome to highlight the importance of community-level screening. We additionally listed a series of secondary outcomes, such as care cascade performance for COPD, asthma, hypertension, and diabetes; health care resource utilization; mental health status; knowledge of various conditions; high-risk behaviors and lifestyles; and socioeconomic profile, to facilitate evaluation of the intervention’s effects across multiple dimensions of population health, including physical, mental, social, and environmental well-being.

Our trial leverages both intrinsic and extrinsic motivation to encourage health workers to support population health. With respect to intrinsic motivation, we provide professional training focused on actively addressing unmet population health care needs. With respect to extrinsic motivation, we incentivize workers through pay-for-population mechanisms that emphasize care cascade indicators. We acknowledge that implementing a new integrated intervention package entails substantial implementation costs, including financial resources as well as the time and effort of primary care providers, and therefore invested in a 3-month program of courses and training in Xishui County covering population medicine theory, survey skills, pulmonary function testing, and communication. Our experience suggests that population medicine and clinical medicine are complementary rather than contrasting. Population-level activities conducted in the community enable the timely detection of previously undiagnosed individuals at high risk of COPD and facilitate their referral for professional clinical management. By structuring these activities as community care delivered by teams of primary care physicians and village health workers, with many tasks (eg, blood pressure and BMI measurement, CBT-based interventions, and smoking cessation interventions) not requiring physicians, the integrated package creates a strong linkage between community and clinical care, supporting earlier and more efficient disease management in low-resource settings.

Our trial has strong policy implications. First, we aim to evaluate whether a multicomponent population medicine intervention is suitable for delivery in community and primary care settings in China. The intervention package represents one potential approach to reforming care at both levels. Second, we seek to encourage policy makers to prioritize population-based screening and long-term management at the community level, rather than focusing primarily on hospital-based treatment. Third, we aim to address unmet health care needs related to tobacco-related NCDs among the high-COPD-risk population, as many high-risk individuals lack awareness of both COPD and common co-occurring conditions, such as asthma, hypertension, and mental health symptoms. Through intensive counseling, health education, and encouragement to seek care, we emphasize the importance of early detection and long-term management in low-resource settings. Fourth, this trial encourages primary and community health care workers to shift from a passive to a proactive mode of practice, in line with the principles of population medicine. Population medicine calls on health professionals to provide care beyond the hospital setting, with community-level screening and referral to the health system playing a central role. Accordingly, this trial asks medical professionals to expand the scope of their responsibilities across multiple dimensions. Specifically, it calls for the provision of holistic care spanning health promotion, screening, disease prevention, diagnosis, treatment, control, and rehabilitation. It also encourages a focus on multimorbidity rather than the separate treatment of single diseases, and an expansion of care delivery beyond hospitals to include communities and populations. This integrated approach may provide valuable guidance for policy development and health care planning. Building on these policy implications, our study further envisions a paradigm shift in the role of primary and community health workers within population medicine.

Specifically, we hope that this study will catalyze a paradigm shift in the role of primary and community health workers. In conventional health systems, frontline providers often operate passively, waiting for patients to present at clinics. This trial introduces mechanisms such as pay-for-population, performance-linked process metrics, and home-based outreach to actively engage providers in population screening, health promotion, and continuous care. This transformation, from a patient-centered, reactive model to a proactive, population-centered approach, lies at the core of population medicine. We believe that this shift has the potential to reshape public health governance not only in China but also in other low- and middle-income countries striving for equitable and sustainable health system reform.

This protocol describes the design and implementation of the POPMIX-COPD trial, the first large-scale, community-based, population medicine–oriented multimorbidity intervention targeting a high-COPD-risk population in China. By integrating population-level screening, proactive disease management, patient education, and performance-linked provider incentives, the study aims to address critical gaps in awareness, early detection, and integrated care for tobacco-related NCDs. Conducted in a resource-constrained setting, this trial has the potential to generate robust evidence for scalable, cost-effective models of multimorbidity management. The findings are expected to inform policy development, improve health care delivery, and promote population health not only in China but also in other low- and middle-income countries striving to achieve equitable and sustainable health system reforms.

## Appendix

Multimedia Appendix 1 Standard Protocol Items: Recommendations for Interventional Trials (SPIRIT) 2025 checklist.

Multimedia Appendix 2 The pay-for-population incentive mechanism.

Multimedia Appendix 3 Secondary outcomes and trial timeline.

Multimedia Appendix 4 Full size versions of Figures 1–3.

## Abbreviations

CBT: cognitive behavioral therapy
COPD: chronic obstructive pulmonary disease
COPD-SQ: COPD Screening Questionnaire
cRCT: cluster randomized controlled trial
DSMB: Data and Safety Monitoring Board
EDC: electronic data capture
FEV1: forced expiratory volume in 1 second
FVC: forced vital capacity
ICC: intraclass correlation coefficient
MDD: minimum detectable difference
NCD: noncommunicable disease
PHQ-9: 9-item Patient Health Questionnaire
POPMIX: Population Medicine Multimorbidity Intervention in Xishui County
SPIRIT: Standard Protocol Items: Recommendations for Interventional Trials
WEMWBS: Warwick-Edinburgh Mental Well-being Scale

## Data Availability

The datasets used or analyzed in this study are available from the principal investigator (CW) on reasonable request.
