# Supplementary material for: Impact of the Population Medicine Multimorbidity Intervention in Xishui County (POPMIX) on People at High Risk for Chronic Obstructive Pulmonary Disease: Protocol for the POPMIX-COPD Cluster Randomized Controlled Trial
Source: JMIR Res Protoc. 2026 Feb 18;15:e85597. doi: 10.2196/85597 (PMC12961387; doi:10.2196/85597)
Supplement: Multimedia Appendix 2 [file resprot_v15i1e85597_app2.docx]

**Design and Implementation of the "Pay-for-Population" Incentive Mechanism**

**in the POPMIX Study**

Many reviewers have drawn attention to the "pay-for-population" motivation mechanism in this study, which involves structuring rewards, funding, motivation intensity, implementation methods, and the role of healthcare workers. To address these concerns and enhance the transparency and replicability of the research, this supplementary material will outline the design, operation process, motivation structure (including both external and internal motivations), funding allocation, and the suitability and risks of implementing this mechanism in resource-constrained areas.

1. **Overall Design Logic of the Pay-for-Population Mechanism**

The POPMIX trial adopts a motivation model that deviates from the traditional "pay-for-performance" approach, which typically centers on clinical outcomes. Instead, it is based on the "pay-for-population" model, where the motivation is linked to population coverage. Traditional "pay-for-performance" models usually tie payments to clinical parameters such as blood pressure, blood glucose control, or emergency exacerbation rates, which are directly associated with clinical outcomes. This approach can inadvertently encourage "selective care" or "managing easier-to-treat populations," resulting in suboptimal motivational responses.[1-3] In contrast, the motivation mechanism in this study is entirely unrelated to clinical outcomes like these, and is instead associated with the intervention coverage related to COPD care at the population level.

In the POPMIX trial, the outcome indicators for the care cascade of township-level clusters (COPD) include screening, diagnosis, treatment, and control management in four phases, with a focus on population coverage, diagnosis rates, treatment rates, and control rates. Of the 1 million yuan allocated for incentive design, 50% will be used for screening, 25% for diagnosis, 15% for treatment, and 10% for control. The indicators for each phase are set within the same framework at the township level for individuals aged 35 and older, with the criteria considering the population's general risks. For example, the screening indicators are based on 13 townships in the county with populations aged 35 and older, reflecting the screening coverage of the high-risk group. The diagnosis phase indicators consider 13 townships and their residents identified as high-risk based on a COPD-SQ score of 16 or above. The treatment phase is based on the 13 intervention townships, with the proportion of confirmed COPD patients receiving standardized inhaled treatment as the denominator.The control phase similarly uses confirmed patients as the denominator and assesses the management coverage of those who did not experience acute exacerbations in the past six months (Table 1).

It is worth emphasizing that these indicators measure whether care is provided, not whether the care is "effective." In other words, this study focuses on ensuring that more residents actually receive basic and necessary chronic disease management, without holding healthcare providers accountable for the physiological disease outcomes of individual patients. This aspect makes the incentive system of this study fairer for grassroots healthcare workers and better suited to resource-limited counties with high levels of complex diseases and heavy care burdens.

**Table 1: Performance Indicators for the Four Stages of the COPD Care Cascade and the Weights in Pay-for-Performance Incentives**

| **Indicator** | **Objective** | **Standard** | **Indicator Weight** |
| --- | --- | --- | --- |
| Screening | Number of individuals aged ≥35 years who completed the initial screening questionnaire (COPD-SQ) in the selected 13 townships / Total number of individuals aged ≥35 years in the 13 townships (%) | In the budget calculation, this project allocates RMB 1,000,000 as incentives for the county hospital (respiratory department), county CDC, and the 13 township health centers. Among them, RMB 500,000 is allocated for screening, RMB 250,000 for diagnosis, RMB 150,000 for treatment, and RMB 100,000 for disease control. | 50% |
| Diagnosis | Number of individuals in the 13 townships who completed spirometry testing / Total number of high-risk individuals who had positive initial screening results in the 13 townships (COPD-SQ >16) (%) |  | 25% |
| Treatment | Number of diagnosed COPD patients in the 13 townships who used inhaled medications / Total number of diagnosed COPD patients in the 13 townships (%) |  | 15% |
| Control | Number of diagnosed COPD patients in the 13 townships who did not experience acute exacerbations in the past 6 months / Total number of diagnosed COPD patients in the 13 townships (%) |  | 10% |

Although the county hospital's respiratory department, the county disease control center, and the 13 township health centers are all included within a unified performance framework, the calculation criteria for incentives differ between county-level institutions and township health centers to ensure that the core content aligns with their respective responsibilities.

***(1) Incentive Calculation Method for Township Health Centers***

The incentives for township health centers are based on the population of their jurisdiction, including residents aged 35 and above, high-risk groups, and already diagnosed patients. The incentive amount is calculated according to the completion rate of the four key indicators at the township level. This approach emphasizes the responsibility of township health centers in community mobilization, high-risk identification, and follow-up management, as well as their coverage efficiency.

***(2) Incentive Calculation Method for County Hospital's Respiratory Department and County Disease Control Center***

The incentives for county-level institutions are calculated based on the overall completion rate of county-wide indicators, reflecting their role in training, quality control, data management, and county-wide coordination. The county hospital's respiratory department is responsible for diagnosis and professional support, while the county disease control center is responsible for regional data aggregation and quality assessment. Using county-wide data ensures that assessment biases due to differences between townships are avoided.

This hierarchical assessment system enables coordinated progress towards common population health goals between county-level institutions and township health centers, while each assumes responsibilities aligned with their functions, achieving organizational collaboration under the framework of population medicine.

Another key advantage of the coverage-based incentive model is that it can proactively expand the reach of healthcare resources. When healthcare workers’ incentives are based on the scope of care coverage rather than individual outcomes, they are more motivated to actively identify and engage with community members who are undiagnosed, untreated, or have not received standardized care. Due to the relatively low marginal cost of community screening and follow-up, once operational workflows are established, 'screening one more person' does not significantly increase workload but helps identify more people with unmet health needs. Therefore, under the advancement of the POPMIX model, the residents actually receiving care often extend beyond the strict boundaries of the research sample, covering a broader population that was previously outside the healthcare system.

1. **The Mechanisms of Incentive Effects on Healthcare Workers: Dual Pathways of Extrinsic and Intrinsic Motivation**

The POPMIX incentive system does not rely solely on financial incentives (extrinsic motivation) to change behavior but combines them with intrinsic motivation to achieve more sustainable behavioral changes in resource-constrained environments.

1. ***Extrinsic Motivation***

At the level of extrinsic motivation, the incentive funds linked to coverage rates provide additional performance resources for grassroots institutions. For township health centers, the incentive amount is directly related to the population size and completion rate in their jurisdiction. This not only sends a clear economic signal at the institutional decision-making level but also transmits through the internal performance distribution mechanisms, motivating frontline healthcare workers to actively participate in community screening, follow-up, and health education activities. The relevant incentives for the respiratory department of the county hospital account for a certain proportion of its annual departmental income, helping to extend the focus of the respiratory specialty from simple outpatient and inpatient care to chronic disease management and capacity building at the county level.

1. ***Intrinsic Motivation***

At the level of intrinsic motivation, the program continuously enhances healthcare workers' professional capabilities in disease management through systematic training, regular quality feedback, and data visualization. The improvement in skills brings a sense of competence, and the autonomy in organizing services at township health centers enhances the sense of work autonomy. The value orientation under the framework of population medicine, which focuses on "providing more residents with the health services they deserve," reinforces a sense of professional mission and achievement. To better understand intrinsic motivation among healthcare workers, the study plans to conduct in-depth interviews to explore how healthcare workers experience and exercise their professional autonomy, sense of competence, and sense of mission in their actual work, and how these factors stimulate their active participation and behavior change in chronic disease management. Interviews will provide a clearer understanding of the specific pathways through which intrinsic motivation functions in the work of grassroots healthcare workers, as well as how the incentive mechanisms affect the sustainability of long-term behavior changes.

The combination of extrinsic and intrinsic incentives provides healthcare workers with both the direct drive of "receiving additional compensation" and the value alignment of "doing the right thing," which is conducive to forming relatively stable behavioral changes.

1. **Impact on Routine Medical Care and Control of Resource Substitution Risks**

In resource-limited county-level environments, the introduction of new incentive mechanisms and interventions may pose a risk of resource substitution, such as reducing the access to care for non-participants or weakening other public health services. To mitigate this risk, during the design phase of POPMIX, there was full communication with the county health bureau to ensure that the project did not increase staffing levels or change the existing outpatient duty system. Additionally, efforts were made to integrate activities such as screening, follow-up, and health education into the current workflow as much as possible, viewing the intervention as a structured and strengthened aspect of daily responsibilities rather than an additional new project. All training and feedback activities were scheduled during regular working hours and did not result in the long-term diversion of healthcare workers from their core clinical tasks.

In the design of POPMIX, the population-based payment incentive mechanism is the central tool for embedding the principles of population medicine into the county-level healthcare system. By linking incentives to the coverage rate of key population segments in areas such as high-risk screening, pulmonary function testing, standardized treatment, and follow-up management—rather than tying incentives to the short-term clinical outcomes of individual patients—this mechanism encourages grassroots healthcare institutions to expand their focus from individual patients to the broader target population of their jurisdiction while fulfilling their existing public health and chronic disease management responsibilities. As a result, healthcare workers no longer assess performance primarily based on "whether a specific patient has been diagnosed and treated," but rather evaluate the system-wide coverage, asking "what proportion of the population in the jurisdiction who should receive screening, diagnosis, and standardized management have actually received these services." This population coverage-based incentive arrangement naturally integrates individual clinical activities into a continuous health management chain aimed at the entire population, reflecting the fundamental approach of population medicine, which combines clinical medicine with public health practices.

1. Eijkenaar, F., *Pay for performance in health care: an international overview of initiatives.* Medical Care Research and Review, 2012. **69**(3): p. 251-276.

2. Mathes, T., et al., *Pay for performance for hospitals.* Cochrane Database of Systematic Reviews, 2019(7).

3. Eijkenaar, F., *Key issues in the design of pay for performance programs.* The European Journal of Health Economics, 2013. **14**(1): p. 117-131.
