# Supplementary figures and images for: Impact of the Population Medicine Multimorbidity Intervention in Xishui County (POPMIX) on People at High Risk for Chronic Obstructive Pulmonary Disease: Protocol for the POPMIX-COPD Cluster Randomized Controlled Trial
Source: JMIR Res Protoc. 2026 Feb 18;15:e85597. doi: 10.2196/85597 (PMC12961387; doi:10.2196/85597)

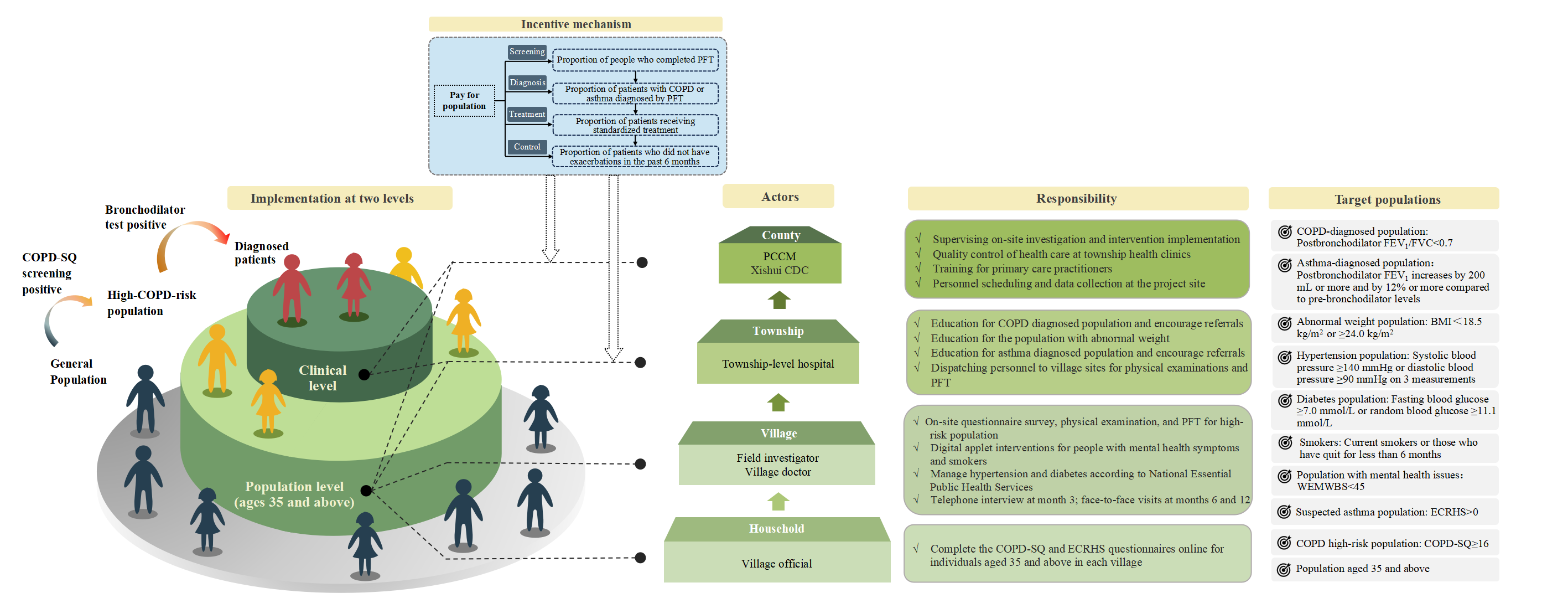

Supplement: Multimedia Appendix 4 [file resprot_v15i1e85597_app4.zip › COPD_Fig1-3/Fig.3.tif]
